# The Role of Genetic and Environmental Factors in White Leg Markings: Prevalence and Heritability Analysis in Pura Raza Española Horses

**DOI:** 10.3390/life15111661

**Published:** 2025-10-23

**Authors:** Ana Encina, María José Sánchez-Guerrero, Manuel Ligero, Arantxa Rodríguez-Sainz de los Terreros, Mercedes Valera

**Affiliations:** 1 Departamento de Agronomía, Escuela Técnica Superior de Ingeniaría Agronómica (ETSIA), Universidad de Sevilla, 41013 Sevilla, Spain; aencina@lgancce.com (A.E.); manololigero72@gmail.com (M.L.); mvalera@us.es (M.V.); 2 Real Asociación Nacional de Criadores de Caballos Españoles (ANCCE), 41014 Sevilla, Spain; arancha@lgancce.com

**Keywords:** coat color, equine, genetic models, genetic parameters, inbreeding, leg marking symmetry, maternal effect

## Abstract

White leg markings in horses are phenotypic traits influenced by genetic and environmental factors. This study analyzed their prevalence, symmetry, and genetic parameters in 38,825 Pura Raza Española (PRE) horses registered in the official studbook. White markings were scored using a binary (affected/unaffected) and a four-level ordinal scale. Most horses (38,341 out of 38,825; 98.8%) had at least one limb without white markings. The prevalence of white markings was higher in the hindlegs (17.9% LH; 14.5% RH) than in the forelegs (5.3% LF; 4.6% RF). Markings were most frequent above the fetlock (≈64%), chestnut horses showed the highest prevalence, whereas black coats showed the lowest. Genetic analyses using Bayesian animal models revealed moderate-to-high heritabilities, ranging from 0.488 for Right hindlegs to 0.574 for Left hindlegs in the multinomial model, which outperformed the dichotomous model (h^2^ = 0.030–0.515 for all legs and left foreleg, respectively). Additive genetic variance was highest in the left hindleg (σ^2^_u_ = 7.904). Genetic correlations were high between contralateral homologous limbs (0.991 forelegs; 0.995 hindlegs), confirming strong bilateral genetic control, while diagonal correlations were lower (≈0.886). These findings confirm a substantial genetic component underlying white leg markings in PRE horses and highlight the importance of refined phenotyping and genetic evaluations for breeding strategies, particularly when markings are penalized as in the PRE breeding program.

## 1. Introduction

White markings and spotting patterns in animal species are thought to result from the domestication process [1]. These white markings, which occur due to the absence of melanocytes in the skin and hair, appear primarily on the legs of horses and are common across various breeds [2]. While the extent and pattern of these markings are largely genetically determined, they are presumably also influenced by stochastic events during embryonic development [3,4]. For instance, melanoblast migration can lead to asymmetry in markings, resulting in differences between the left and right legs, or between the fore and hind legs. The observation (mainly in horse clones used in Polo) that cloned horses frequently exhibit differing white markings distribution despite having identical genotypes further supports the contribution of non-genetic, developmental factors to phenotypic variability. This interplay of genetic, environmental, and developmental factors complicates the prediction of white markings, even when the genetic background is well understood. The emphasis on symmetry in white leg markings in horses is primarily related to aesthetic preferences in various breeds and morphological competitive settings. Symmetrical markings often create a balanced and harmonious appearance, which is highly valued in many breed standards and by show judges. However, these markings have no proven link to performance or athletic ability, and many top-performing horses exhibit asymmetrical markings without any impact on their capabilities. The focus on symmetry is therefore more about adhering to breed conformation standards, which are outlined by associations to guide breeders in producing horses that exemplify the “ideal” image of a particular breed.

The inheritance of white markings in horses is a complex, multifactorial trait influenced by a combination of genetic and environmental factors. Studies on Arabian horses have shown that these markings are controlled by both major and minor genes [3,5]. These genetic variants are responsible for controlling melanocyte migration and proliferation during early development, leading to the observed variation in white markings on the body and leg [5].

Several loci have been identified as key contributors to this phenotype, with genes such as KIT and MC1R playing a significant role in the presence and distribution of white markings [6,7,8,9]. These highlight a strong genetic component underlying these particular phenotypes. Similar findings have been reported in other breeds, such as German sport horse populations [10]. Additionally, research in the Swiss Franches-Montagnes Horse Breed revealed high heritabilities and genetic correlations for white leg markings [1]. A strong positive correlation was found between the chestnut allele at the melanocortin-1-receptor gene locus and the extent of white markings.

The aforementioned genetic and phenotypic variability across different breeds highlights the importance of understanding these traits within specific populations. The Pura Raza Española Horse (PRE), as the main breed in Spain, stands as a key example. It is one of the most important equine breeds worldwide, both in terms of census and influence on other breeds. It has played a key role in the development of numerous American and European breeds, such as the Lusitano and the American Quarter Horse [11]. Known for its elegance, strength, and versatility, the PRE horse is highly valued both for leisure riding and for competitive disciplines such as Dressage [12]. Its genetic legacy and adaptability have made the PRE one of the most sought-after breeds in the equine industry, being bred in more than 67 countries [13]. The genetic diversity of the PRE breed also includes a range of coat colors, with white spots on the legs being a prominent feature in some individuals [14]. Understanding the genetic and environmental basis of these markings is crucial not only for the PRE but also for other breeds where aesthetics are a major selection criterion, particularly where extensive or asymmetrical white markings are penalized.

The aim of this study was to determine the prevalence of white leg markings in Pura Raza Española horses, examining their relationship to coat color. Additionally, this research will assess the symmetry of white markings, both laterally and anteroposteriorly, and explore the genetic parameters of these traits.

## 2. Materials and Methods

### 2.1. Description of the Traits and Database

To carry out this study, a database of 38,825 horses was analyzed to determine the number and proportion of PRE horses affected by white markings on their legs, according to their degree. The average evaluation age of the horses was over six months (the data were collected when the grey horses were foals, so they still had a “no white” coat color, allowing white markings to be clearly identified). These records were taken in horses born between 2005 and 2021, from Spain, during the systematic veterinarian evaluation that all horses must undergo before being registered in the PRE [15].

For this study, white markings on legs were analyzed using two approaches (Table 1):

i. Dichotomous trait: classified as unaffected (class 0) or affected (class 1).
ii. Discrete scale: a four-level scoring system (class 0: unaffected; class 1: white markings below the fetlock; class 2: markings above the fetlock; class 3: markings up to the cannon bone). The scale’s extremes represented the biological limits of this trait.

In addition to studying the prevalence and heritability of the four legs, the laterality, symmetry, and diagonality of the white marks with the following combinations were studied:

A. Left foreleg + Right foreleg: (LF) + (RF);
B. Left hindleg + Right hindleg: (LH) + (RH);
C. Left legs: (LF) + (LH);
D. Right legs: (RF) + (RH);
E. Left foreleg + Right hindleg: (LF) + (RH);
F. Right foreleg + Left hindleg: (RF) + (LH);
G. Four legs: (LF) + (RF) + (LH) + (RH).

### 2.2. Statistical and Genetic Model

All statistical analyses were performed using the R statistical software (version number 4.3) environment [16]. A Bayesian approach was employed for statistical analysis, primarily utilizing the BRMS package [17] to fit a generalized multivariate (linear) multilevel model.

The prevalence of white markings on the legs of PRE horses and their proportion according to the degree of these markings were first determined.

A cumulative link model was used for the ordinal outcomes of the discrete scale analysis. The model was defined as follows:

$$log\left(\frac{P(Y\leq j)}{1-P(Y\leq j)}\right)=\alpha_{j}+\beta_{1}X_{1}+\beta_{2}X_{2}+\cdots +\beta_{k}X_{k}$$

where P(Y≤j) is the cumulative probability of an observation falling into category j or below; $\alpha_{j}$ is the intercept parameter for each cumulative logit; and $\beta_{k}$ is the coefficient for the *k*-th covariate ($X_{k}$).

The potential systematic risk factors studied were *sex* (2 levels: male, *n* = 13,166; and female, *n* = 25,659) and *coat color* (4 levels: grey, *n* = 15,681; bay, *n* = 15,011; chestnut, *n* = 3292; and black, *n* = 4841).

The potential covariate factors studied were as follows:

The pedigree-based inbreeding coefficient (F), calculated using the complete genealogical information available in the PRE studbook, was treated as a continuous variable ranging from 0.001 to 0.429, with an average of 0.074 (SD 0.045).

A maternal random effect (23,242 levels) was also included in the model to separate its influence from the direct additive genetic effect.

A multivariate animal model was used to estimate genetic parameters for white markings on the leg, analyzing them as both a binomial trait (affected or not affected) and discrete traits (using a four-score scale for dimensions). The models included systematic effects of all influencing factors whose 95% credible intervals (CrI) excluded zero in the previous statistical analysis: sex, coat color, inbreeding, and maternal effects.

Additive genetic and residual effects were included as random factors. The genetic parameters for both models were estimated using a Bayesian approach via Gibbs sampling. All analyses were performed with the GIBBSF90+ module of the BLUPF90 software (version 3.16) [18]. The Gibbs sampler was run for 100,000 rounds. Convergence was assessed by examining trace plots, and the first 10,000 iterations were discarded as burn-in to ensure stable and reliable estimates. Subsequently, every 100th sample was saved for later analysis. Posterior means and standard deviations were calculated with the POSTGIBBSF90 software (version 3.15) [18] to obtain estimates of variance components.

The equation in matrix notation to solve the mixed model was as follows:

y = X_b_ + Z_u_ + W_m_ + e with

$$\left[\begin{array}{c} u\\ m \end{array}\right]\;\sim \;N\left(0,\;\left[\begin{array}{cc} A\sigma_{u}^{2} & {A\sigma }_{um}\\ {A\sigma }_{um} & A\sigma_{m}^{2} \end{array}\right]\right)\;e\;\sim \;N(0,\;I\sigma_{e}^{2})$$

where y is the vector of observations, X is the incidence matrix of systematic effects, Z is the incidence matrix of animal genetic effects, W is the incidence matrix of maternal genetic effects, b is the vector of systematic effects, u is the vector of direct animal genetic effects, m is the vector of maternal genetic effects, e is the vector of residuals, σ_u_^2^ is the direct genetic variance, σ_m_^2^ is the maternal genetic variance, σ_e_^2^ is the residual genetic variance, I is an identity matrix, and A is the numerator relationship matrix.

In order to identify the precision of the parameters, the 95% highest posterior density (HPD) intervals were determined from their marginal posterior distributions. ENDOG software (version number V4.8) [19] was used to estimate the inbreeding coefficient of animals.

The pedigree information necessary for genetic evaluation was obtained from the official PRE studbook, including all available generations and comprising a total of 83,334 horses.

Table 2 presents the number and proportion (%) of Pura Raza Española (PRE) horses categorized by white leg marking scores. Most horses were unaffected, with the highest proportion of unaffected horses observed in the combination of all four legs (98.75%, combination G). The prevalence of white markings varied significantly across anatomical regions and specific limbs. A clear difference was observed between forelegs and hindlegs. The prevalence of markings on the hindlegs was substantially higher, ranging from 14.51% (right hindleg) to 17.95% (left hindleg), compared to the forelegs, which showed a lower prevalence of 4.65% (right foreleg) to 5.29% (left foreleg). This asymmetry was further highlighted by the fact that only 1.25% of the evaluated horses had white markings on all four legs, while the prevalence of markings on both left legs (combination C) and both hindlegs (combination B) was 3.51% and 9.15%, respectively.

Among the affected limbs, the distribution of markings by score also showed a clear and consistent pattern. White markings were most frequently observed in the “above fetlock” category, ranging from 63.79% to 66.16% depending on the specific limb. In contrast, markings in the “below fetlock” were the least common, with a frequency ranging from 12.14% to 16.91%.

The associations between systematic risk factors and white leg marking scores in Pura Raza Española (PRE) horses are summarized in Tables S1 and S2. The results of the Bayesian multivariate models reveal significant effects of several systematic factors, particularly coat color, sex, and, importantly, maternal effects, on the presence and extent of white markings. All the analyzed risk factors, with the exception of inbreeding for certain traits, were found to be statistically relevant, as their 95% credible intervals did not include zero.

For all four limbs (LF, RF, LH, and RH), coat color consistently showed a statistically relevant effect. Chestnut horses showed the highest prevalence of white markings, while black horses had the lowest. For example, in the Left foreleg, 91.98% of chestnut horses had unmarked legs, compared to 94.36% of grey and 96.57% of black horses.

The sex of the horse also influenced white markings, particularly in the forelegs, where females had a higher proportion of unmarked legs than males. Specifically, in the LF, 95.45% of females lacked white markings versus 93.26% of males. This pattern was observed across all limbs, though it was less pronounced in the hindlegs.

The effect of inbreeding was statistically relevant only in the forelegs (LF and RF), as the credible intervals (0.25, 2.27) and (0.02, 2.23), respectively, did not include zero. This indicates a positive association between the inbreeding coefficient and the probability of having white markings in these limbs. In contrast, for the hindlegs (LH and RH), the intervals (e.g., −0.62, 0.74) did include zero, suggesting no relevant association.

The maternal effect (variance due to the dam) was consistently relevant across all limbs, with 95% credible intervals that did not include zero, such as (0.91, 1.19) in the LF and (0.82, 0.98) in the RH. This indicates that maternal identity contributes significantly to the variability in white leg markings, possibly reflecting maternal genetic background or early developmental factors.

In summary, coat color, sex, and maternal effects were the most influential factors affecting white markings. The effect of inbreeding and maternal factors, however, showed limb-specific patterns, being stronger in forelegs and weaker or absent in hindlegs.

The genetic parameters estimated in this study are presented in Table 3 and Table 4, which correspond to different modeling approaches. Table 3 provides results for the dichotomous model (two classes: affected vs. not affected), while Table 4 corresponds to the multinomial ordinal model with four classes (0–3). These models were used to estimate the additive genetic variance (σ^2^_u_), maternal genetic variance (σ^2^_m_), residual variance (σ^2^_e_), and heritability (h^2^) for white leg marking scores. The HPD95% intervals represent the range within which the true parameter values are likely to fall with 95% probability, offering insights into the precision and variability of the estimates.

Genetic parameter estimates from the dichotomous model (Table 3) revealed substantial differences in the genetic control of white markings across individual legs and their combinations. The additive genetic variance (σ^2^_u_) was found to be the primary source of variability, while the maternal variance (σ^2^_m_) was consistently small and stable for individual legs (mean σ_m_ ranging from 0.037 to 0.067). This suggests that maternal effects have a limited influence on the presence of white markings on a single limb. In contrast, the maternal variance became more pronounced in certain leg combinations, particularly for categories B (LH + RH) and F (RF + LH), where the mean σ_m_ values were considerably higher (1.788 and 2.833, respectively). However, the wide HPD95% intervals associated with these estimates (e.g., 0.007–6.721 for combination B) indicate greater uncertainty in the estimation of maternal effects for these more complex, multi-leg traits.

Heritability estimates highlight these differences in genetic control. On the underlying scale (h^2^), which reflects the genetic control of a continuously expressed trait, heritabilities were moderate to high, ranging from 0.394 (for combination G) to 0.645 (RF). Notably, heritabilities for the individual forelegs (LF: 0.591; RF: 0.645) were consistently higher than those for the hindlegs (LH: 0.539; RH: 0.525). This finding contrasts with the observed heritability (h^2^observed), which shows a different trend within the same table. Heritabilities on the observed scale (h^2^_observed_), which represent the proportion of phenotypic variance on the binary scale, were significantly lower. They ranged from a very low 0.030 (combination G) to a maximum of 0.515 (LH). This stark difference between the two scales indicates that while a substantial underlying genetic control exists, the binary manifestation of the trait as “affected” or “unaffected” results in reduced observable heritabilities, especially for complex multi-leg patterns.

In the multinomial ordinal model (Table 4), genetic parameters were estimated for individual legs based on the four-level scoring system. The results revealed clear patterns in the distribution of genetic variance. The estimates were generally precise, as indicated by the relatively narrow HPD95% intervals for most parameters.

Hindlegs consistently showed higher additive genetic variance (σ^2^_u_) compared to the forelegs, with the left hindleg having the highest mean (σ^2^_u_ = 7.904) and the right hindleg having a mean of 5.659. In contrast, forelegs showed lower additive genetic variance (LF: 4.877; RF: 4.772).

A similar trend was observed for maternal genetic variance (σ^2^_m_), which was also higher in the hindlegs (LH: 0.398; RH: 0.273) compared to the forelegs (LF: 0.185; RF: 0.130). Although generally low, these higher maternal estimates in hindlegs suggest that non-additive effects may be somewhat more relevant in these limbs, though their HPD95% intervals indicate a wider range of uncertainty compared to the additive variance estimates.

Heritability estimates (h^2^) from the multinomial ordinal model, while representing the proportion of total variance attributable to additive effects, also showed a distinct pattern. The left hindleg had the highest heritability at 0.574 (SD: 0.029), followed by the right foreleg at 0.539 (SD: 0.028). The right hindlegs had the lowest heritability (0.488; SD: 0.043) among the four limbs.

Genetic correlations between white markings on four limbs are presented in Table 5 and reveal strong relationships in the expression of this phenotype. These correlations were high and consistent across both the dichotomous and multinomial models, indicating a substantial degree of shared genetic control.

The highest correlations were consistently observed between contralateral homologous limbs. In the multinomial model (values above the diagonal), the genetic correlation between the left and right forelegs was extremely high at 0.991 (SD = 0.003; HPD95%: 0.985–0.995), while the correlation between the left and right hindlegs was 0.995 (SD = 0.002; HPD95%: 0.991–0.998). These results indicate that the genetic control of white markings exhibits a high degree of symmetry between bilateral limb pairs.

Substantial correlations were also found between non-homologous limbs, although they were slightly lower. In the multinomial model, correlation between fore and hindlimbs ranged from 0.863 to 0.907. For example, the correlation between the left foreleg and left hindleg was 0.907 (SD = 0.014; HPD95%: 0.883–0.936). Diagonal correlations, such as between the left foreleg and right hindleg (0.863; SD = 0.019; HPD95%: 0.823–0.899) and the right foreleg and left hindleg (0.887; SD = 0.026; HPD95%: 0.840–0.934), were also high but consistently the lowest among all limb pairs, reflecting an expected decrease in genetic symmetry across diagonals axes.

The dichotomic model (values below the diagonal) showed a similar overall pattern to the multinomial model, but with slightly lower correlation values. For instance, the genetic correlation between the left and right forelegs was 0.988 (SD = 0.005; HPD95%: 0.977–0.995), and between the left and right hindlegs, 0.986 (SD = 0.005; HPD95%: 0.975–0.993). Correlations between fore and hindlimbs ranged from 0.866 to 0.887, confirming a moderate to high level of shared genetic control between front and back legs.

## 3. Discussion

The study of white leg markings in horses is relevant for understanding the hereditary and phenotypic expressions that influence breeding programs and animal health. White markings, particularly when associated with pink skin, may increase the risk of photosensitivity, dermatitis, and other skin pathologies due to UV exposure [20]. In addition, depigmentation phenotypes often arise from pleiotropic effects of pigmentation genes, which may have broader implications for animal health, as has been described in horses [21]. These depigmentation phenotypes and color variation are thought to be a result of domestication processes, which are sometimes accompanied by behavioral changes, such as tamability [22,23,24,25]. While direct associations between white markings and performance characteristics such as temperament have yet to be fully explored, there is ongoing interest in how these phenotypic traits may correlate with behavioral and physical attributes in certain competitive settings. In the context of breed management and market value, the presence of these markings is especially relevant in breeds such as the PRE; excess whiteness on the legs can disqualify an individual from breeding programs [14]. Therefore, understanding heritability and the factors that contribute to white markings offers valuable tools for maintaining breed integrity.

The present study provides novel insights into the distribution and prevalence of white leg markings in PRE horses. The observed prevalence ranged from a low of 1.25% for horses with markings on all four legs to 17.95% on the left hindleg alone. These findings suggest a moderate frequency of white markings in this breed when compared to other equine populations. For instance, studies in Swiss Franches-Montagnes horses reported high heritability and notable prevalence of leg markings [1]. Similarly, Quarter Horses documented substantial variability in the extent of white markings, emphasizing their genetic complexity [2]. Our results indicate a lower overall prevalence, particularly in the forelegs, which aligns with findings in other breeds and may reflect selection preferences within the PRE breeding program, where extensive white markings are penalized [14].

Interestingly, the lower prevalence of foreleg markings observed in our study mirrors trends reported in Quarter Horses, where forelegs are less commonly affected compared to hindlegs [2]. This difference might reflect variations in the genetic regulation of pigmentation pathways across limb regions, potentially involving distinct developmental mechanisms. This asymmetry is particularly relevant in the context of breed standards, where white markings are often viewed as undesirable and can lead to disqualification if they exceed specific thresholds [14,26]. For example, in the Pura Raza Menorquina, excessive white markings are penalized to maintain the breed’s solid black coat [27], while in the Swiss Franches-Montagnes, selection favors minimal white [1]. Conversely, for breeds like the American Paint Horse, a white spotting phenotype is a prerequisite for registration in the “Regular” registry, which significantly impacts the horse’s economic value [28,29]. Our results provide a foundation for understanding the genetic basis of this phenotypic asymmetry, which is critical for breeds like the PRE, where excessive white markings can lead to exclusion from the studbook [14].

Genomic studies targeting pigmentation-associated loci, combined with historical records of breeding practices, could provide deeper insights into the evolutionary and functional significance of these traits. In this study, the prevalence of white markings in PRE horses exhibits significant associations with coat color, a trend consistent with studies in other breeds. Our study revealed a notably higher prevalence of white markings in hindlegs (LH: 17.95%, RH: 14.51%) compared to forelegs (LF: 5.29%, RF: 4.65%), particularly in darker coat colors such as black and bay, where markings tend to be less common. Similar findings have been reported in Swiss Franches-Montagnes horses [1], where chestnut-coated individuals exhibited significantly larger and more frequent white markings than darker-coated counterparts. These observations suggest a pleiotropic effect of coat color genes, such as *MC1R* and *ASIP*, influencing both pigmentation and white marking expression. The relationship between coat color and white markings may be explained by genetic variants at loci such as KIT and PAX3, which are associated with melanocyte survival and migration during development [6,7]. The impact of coat color on the prevalence of white markings has also been studied in PRE horses, where chestnut-coated individuals showed the highest prevalence of white markings (57.3% affected), while black-coated horses showed the lowest (29.4% affected) [4].

Beyond the biological implications, coat color carries substantial weight in the equine sector due to its influence on marketability, aesthetic preferences, and breed traditions. In the PRE horse, while the grey phenotype is predominant and historically favored for its association with nobility and tradition [11], emerging international markets are increasingly drawn to bay and black individuals. However, these darker coats tend to be less associated with white markings. Therefore, it could be expected that, if this trend continues, the presence of white markings in the offspring will decrease. Understanding the interaction between coat color and white markings thus holds dual importance: biologically for deciphering pigmentation genetics, and economically for guiding breeding strategies aligned with market demand.

In our study, we have analyzed the influence of not only sex and coat color but also the inbreeding coefficient and the maternal effect as environmental factors (Supplementary Tables S1 and S2). Sex was found to be significantly associated with the presence of white leg markings, with males showing a higher prevalence than females. This trend has also been observed in Arabian horses, where a potential hormonal influence or differential selective pressures between sexes was suggested [3]. In the context of the PRE breed, aesthetic preferences and registration requirements may lead to differential retention of males and females in breeding programs, thus impacting the observed prevalence. These findings reinforce the importance of considering sex in genetic evaluations of phenotypic traits.

The inbreeding coefficient had no statistically significant effect on most traits, except for the forelegs, where a modest influence was observed. This result is consistent with findings in the Franches-Montagnes breed, where no strong association between inbreeding and white markings was identified [1]. The limited variability in inbreeding levels in our population (mean F = 0.074) may explain the lack of a robust effect. Overall, this suggests that specific loci, rather than genome-wide homozygosity, predominantly influence the presence of white markings.

In general, all these effects were statistically significant for most of the traits analyzed. Some of these environmental effects have been used previously in this equine breed to estimate genetic parameters related to morphological defects such as ewe neck [30], cresty neck [15], or limbs defects [31]; those related to pathologies such as the presence of skin melanomas [32] or obesity [33]; and those related to the evaluation of morphological [34], behavioral [35], reproductive [36], or performance variables [12].

The heritability estimates for white markings obtained in this study are consistent with previous findings across different breeds, confirming a relevant genetic basis for these traits [1,5]. A key observation concerns the difference between the two models employed: the multinomial ordinal model (Table 4), which incorporated a four-level scoring system, yielded moderate-to-high heritabilities (h^2^ = 0.488–0.574), whereas the dichotomous model (Table 3) produced markedly lower estimates (h^2^ = 0.030–0.515). This difference highlights the importance of using more detailed scoring systems, as dichotomous classifications tend to underestimate the genetic variability by oversimplifying the trait into “affected” versus “not affected.” Comparable values for white markings were reported in the Pura Raza Menorquina, where heritability estimates based on three categories (few, medium, many) reached 0.23. Notably, this breed allows only black-coated animals in its studbook, making the management of white markings a critical breeding goal [27].

Our findings for white markings are also in agreement with previous studies, which reported high heritability in Arabian horses (h^2^ = 0.68) and similar estimates in the Swiss Franches-Montagnes (h^2^ = 0.52 for forelimbs, h^2^ = 0.58 for hindlimbs) [1,5]. More recently, moderate-to-high heritabilities for white markings depending on coat color were confirmed, with values ranging from 0.32 in chestnut, 0.47 in bay, 0.53 in black, and 0.40 in grey when modeled linearly [4]. These results support the view that markings are under a strong but complex genetic control, influenced both by the base coat color and by the genetic model applied.

Within the multinomial model, a noteworthy pattern also emerged regarding the genetic control of individual limbs. While the heritability estimates were moderate to high overall, a clear asymmetry was observed: the left hindleg showed the highest heritability (h^2^ = 0.574), whereas the right hindleg had the lowest (h^2^ = 0.488), despite both having high additive genetic variance. This apparent contradiction is explained by the higher residual variance in both hindlegs, which diluted the heritability, especially in RH. In contrast, the extremely high additive variance in LH (7.904) was sufficient to overcome the high residual variance, resulting in the highest heritability observed. Taken together, these moderate-to-high heritability estimates, combined with the very high genetic correlations found between limbs (Table 5), suggest that selection for white markings would be highly effective. The strong correlations, particularly within homologous limb pairs, indicate that the genetic control for markings is largely shared across all four limbs, consistent with the symmetrical regulation of white markings and further corroborated in the Franches-Montagnes breed [1,3]. Moreover, the influence of coat color on the expression of white markings suggests that both base pigmentation and developmental processes can modulate the extent of white markings, reinforcing the need for refined phenotyping and multivariate evaluations to effectively manage this trait while maintaining breed standards and genetic diversity [4].

The strong genetic correlations detected between limbs (Table 5) closely mirror the phenotypic symmetry observed in the PRE population, confirming that the genetic architecture of white markings strongly favors bilateral expression. This pattern was consistent across both models, with the multinomial model generally showing slightly higher values due to its more detailed scoring system. Correlations were highest between contralateral homologous limbs (r = 0.995 for hindlegs; r = 0.991 for forelegs in the multinomial model), and slightly lower for diagonal pairs (e.g., r = 0.886), suggesting that the regulation of white markings is bilaterally symmetrical and largely shared across limb pairs. Similar findings have demonstrated that both directional and anteroposterior asymmetries of white leg markings have a genetic basis, with greater genetic variation associated with anteroposterior asymmetry [3], and that the genetic architecture of white markings in Franches-Montagnes horses strongly favors bilateral expression [1]. The alignment between phenotypic and genetic correlations underscores the heritable nature of limb-specific pigmentation patterns, while also indicating that developmental factors may contribute to the observed variability. Although genetic control plays a predominant role, stochastic events during embryonic development can affect melanocyte migration and survival, leading to asymmetries that generate part of the phenotypic variation [3]. Taken together, these findings support the conclusion that white leg markings are under strong and symmetrical genetic regulation, particularly within limb pairs, and to a lesser extent across the sagittal and diagonal axes.

These findings have important practical implications for breeding programs. The moderate-to-high heritability estimates, combined with the very high genetic correlations, suggest that selection for white markings would be highly effective. In particular, the strong genetic correlations observed between symmetrical limbs imply that selection against markings on a specific limb would likely produce correlated responses in its contralateral counterpart, highlighting the need for careful multivariate evaluations to minimize the trait without compromising genetic diversity. Since excessive white markings are considered a disqualifying trait in the PRE breeding program [14], these insights provide valuable tools for breeders. More broadly, the evidence confirms that white markings in horses are influenced by a polygenic system with moderate-to-high heritability and strong genetic correlations across homologous regions. The consistency between our results and those from other breeds [1,3,4,5,27], as well as within the same breed for facial markings [4], supports the potential for efficient selection against (or in favor of) these traits. Furthermore, the higher resolution provided by multinomial and linear models underscores the importance of refined phenotyping approaches to capture the underlying genetic variability, particularly when coat color heterogeneity interacts with the expression of markings.

## 4. Conclusions

White leg markings in Pura Raza Española horses result from the complex interplay between genetic and environmental factors, with clear and consistent effects of coat color, sex, inbreeding, and especially the maternal effect. From the genetic perspective, heritability estimates were moderate to high, especially in hindlegs, suggesting ample genetic variability that could be harnessed through selective breeding. The presence of high genetic correlations between contralateral limbs indicates that white markings are symmetrically inherited, particularly within forelegs and hindlegs. The results of the Bayesian multivariate models confirm that the maternal effect is statistically relevant across all limbs. This highlights the need to account for mare identity in genetic evaluations, either due to maternal genetic transmission or early developmental influences. In contrast, inbreeding showed a statistically significant effect only on the forelegs, with negligible impact on hindlegs. Overall, these findings reinforce the multifactorial nature of white leg markings in Pura Raza Española horses. The identification of relevant genetic parameters, along with environmental contributions, provides a robust basis for informed selection strategies. Given that extensive white markings are considered a disqualifying trait in the Pura Raza Española breed standard, genetic selection can be effectively used to minimize their expression, while maintaining genetic diversity and respecting morphological integrity. Further genomic studies will be needed to identify specific loci involved in these pigmentation traits and to support the implementation of precise breeding tools for managing white markings in this iconic breed.

## Figures and Tables

**Table 1:** Description of the scoring system for white leg markings in the Pura Raza Española horse.

	No Affected	Below Fetlock	Above Fetlock	Cannon Bone
Foreleg	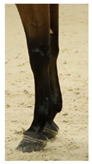	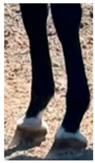	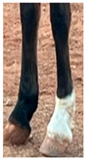	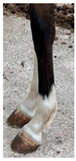
Hindleg	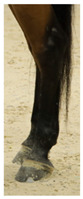	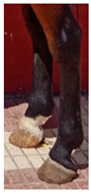	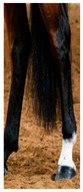	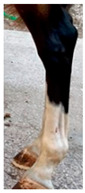

**Table 2:** Number and proportion (%) of Pura Raza Española horses by white leg marking scores.

White Leg Markings	Not Affected		Affected		Below Fetlock		Above Fetlock		Cannon Bone	
LF	36,771	(94.7)	2054	(5.3)	332	(16.2)	1359	(66.2)	363	(17.6)
RF	37,021	(95.4)	1804	(4.6)	305	(16.9)	1172	(65.0)	327	(18.1)
LH	31,857	(82.1)	6968	(17.9)	846	(12.1)	4451	(63.9)	1671	(24.0)
RH	33,191	(85.5)	5634	(14.5)	714	(12.7)	3594	(63.8)	1326	(23.5)
A	37,928	(97.7)	897	(2.3)						
B	35,273	(90.8)	3552	(9.2)						
C	37,462	(96.5)	1363	(3.5)						
D	37,736	(97.2)	1089	(2.8)						
E	37,644	(97.0)	1181	(3.0)						
F	37,621	(96.9)	1204	(3.1)						
G	38,341	(98.8)	484	(1.2)						

LF: left foreleg; RF: right foreleg; LH: left hindleg; RH: right hindleg; A: (LF) + (RF); B: (LH) + (RH); C: (LF) + (LH); D: (RF) + (RH); E: (LF) + (RH); F: (RF) + (LH); G: (LF) + (RF) + (LH) + (RH).

**Table 3:** Genetic parameters (mean and median for additive genetic variance (σ^2^_u_), maternal variance (σ^2^_m_), and residual variance (σ^2^_e_) and heritabilities (h^2^) for white markings score in leg in a dichotomous model.

White Leg Markings		σ^2^_u_			σ^2^_m_			σ^2^_e_		h^2 (1)^	h^2^_observed_^(2)^
	Mean	Median	HPD 95%	Mean	Median	HPD 95%	Mean	Median	HPD 95%		
LF	1.540	1.531	1.280–1.805	0.059	0.055	0.019–0.111	1.000	1.000	0.978–1.020	0.591 (0.022)	0.187
RF	1.945	1.938	1.594–2.279	0.067	0.061	0.010–0.140	1.000	1.000	0.978–1.021	0.645 (0.022)	0.180
LH	1.223	1.218	1.06–1.396	0.043	0.041	0.018–0.071	1.000	1.000	0.979–1.018	0.539 (0.017)	0.515
RH	1.150	1.145	1.003–1.309	0.037	0.036	0.013–0.056	1.000	1.000	0.978–1.020	0.525 (0.017)	0.418
A	1.555	1.350	0.120–3.221	0.610	0.490	0.009–2.103	1.016	1.001	0.796–1.209	0.472 (0.149)	0.101
B	4.133	2.165	0.462–13.750	1.788	0.404	0.007–6.721	1.013	1.000	0.673–1.356	0.572 (0.102)	0.098
C	1.687	1.517	0.042–3.940	0.402	0.234	0.002–1.401	1.168	1.005	0.439–2.387	0.495 (0.177)	0.070
D	1.975	1.332	0.040–5.173	0.961	0.268	0.013–4.033	1.391	1.008	0.714–3.100	0.465 (0.130)	0.245
E	1.002	0.986	0.058–1.903	0.272	0.058	0.004–1.300	1.007	1.001	0.747–1.167	0.410 (0.187)	0.076
F	4.957	2.083	0.841–21.230	2.833	0.613	0.008–16.320	0.977	0.998	0.467–1.353	0.573 (0.075)	0.108
G	1.161	0.983	0.421–2.461	0.382	0.211	0.011–1.371	1.150	1.007	0.522–2.248	0.394 (0.209)	0.030

^(1)^ Heritability (h^2^) underlying scale; ^(2)^ heritability real scale (h^2^_observed_); HPD95%: highest posterior density at 95, intervals represent the range within which the true parameter values are likely to fall with 95% probability; σ^2^_u_: additive genetic variance; σ^2^_m_: maternal variance; σ^2^_e_: residual variance; LF: left foreleg; RF: right foreleg; LH: left hindleg; RH: right hindleg; A: (LF) + (RF); B: (LH) + (RH); C: (LF) + (LH); D: (RF) + (RH); E: (LF) + (RH); F: (RF) + (LH); G: (LF) + (RF) + (LH) + (RH).

**Table 4:** Genetic parameters (mean and median for additive genetic variance (σ^2^_u_), residual variance (σ^2^_e_), and heritabilities (h^2^) for white markings score in leg in a four-class multinomial model.

Genetics Parameters		Left Foreleg (LF)	Right Foreleg (RF)	Left Hindleg (LH)	Right Hindleg (RH)
	Mean	4.877	4.772	7.904	5.659
σ^2^_u_	Median	4.871	4.781	7.922	5.657
	HPD 95%	4.341–5.368	4.188–5.243	7.060–8.719	4.971–6.218
	Mean	0.185	0.130	0.398	0.273
σ^2^_m_	Median	0.181	0.121	0.400	0.271
	HPD 95%	0.062–0.309	0.014–0.273	0.119–0.589	0.146–0.410
	Mean	4.000	3.420	8.046	5.898
σ^2^_e_	Median	3.968	3.411	7.794	5.730
	HPD 95%	3.295–4.611	2.868–3.966	5.292–11.280	4.036–7.928
h^2^		0.514 (0.061)	0.539 (0.028)	0.574 (0.029)	0.488 (0.043)

σ^2^_u_: additive genetic variance; σ^2^_m_: maternal variance; σ^2^_e_: residual variance; h^2^: heritability; HPD95% intervals represent the range within which the true parameter values are likely to fall with 95% probability.

**Table 5:** Genetic correlations and standard deviation (SD) between the four legs for the presence of white markings in the Pura Raza Española horses dichotomous (below the diagonal) and multinomial (above the diagonal).

White Leg Markings	Left Foreleg (SD) [HPD 95%]	Right Foreleg (SD) [HPD 95%]	Left Hindleg (SD) [HPD 95%]	Right Hindleg (SD) [HPD 95%]
Left Foreleg		0.991 (0.003)[0.985–0.995]	0.907 (0.014) [0.883–0.936]	0.886 (0.016) [0.854–0.915]
Right Foreleg	0.988 (0.005) [0.977–0.995]		0.887 (0.015) [0.854–0.915]	0.863 (0.019) [0.823–0.899]
Left Hindleg	0.887 (0.026) [0.840–0.934]	0.866 (0.021) [0.828–0.909]		0.995 (0.002) [0.991–0.998]
Right Hindleg	0.883 (0.019) [0.848–0.920]	0.858 (0.020) [0.820–0.896]	0.986 (0.005) [0.975–0.993]	

HPD95% intervals represent the range within which the true parameter values are likely to fall with 95% probability; SD: standard deviation.

## Data Availability

Data are available at 10.5281/zenodo.16893897.

## Supplementary Materials

The following supporting information can be downloaded at: https://www.mdpi.com/article/10.3390/life15111661/s1, Table S1: Bayesian generalized multivariate (non-)linear multilevel models and “relevant” level (when the 95% credible interval does not include zero and are highlighted in grey) between white markings in leg score (discrete scale) and systematic risk factors and tests for comparing proportions between groups for discrete systematic risk factors; Table S2: Bayesian generalized multivariate (non-)linear multilevel models and “relevant” level (when the 95% credible interval does not include zero and are highlighted in grey) between white markings in leg score (Dichotomous trait) and systematic risk factors and tests for comparing proportions between groups for discrete systematic risk factors.

## Abbreviations

The following abbreviations are used in this manuscript:

PRE	Pura Raza Española
ANCCE	Real Asociación Nacional de Criadores de Caballos Españoles
BLUPF90	Best Linear Unbiased Prediction FORTRAN 90
LF	Left foreleg
RF	Right foreleg
LH	Left hindleg
RH	Right hindleg
A	Left foreleg + Right foreleg
B	Left hindleg + Right hindleg
C	Left foreleg + Left hindleg
D	Right foreleg +Right hindleg
E	Left foreleg + Right hindleg
F	Right hindleg + Left hindleg
G	Left foreleg + Right foreleg + Left hindleg+ Right hindleg
ENDOG	Computer program for analyzing pedigree information
HPD95%	Intervals represent the range within which the true parameter values are likely to fall with 95% probability
SD	Standard deviation
CrI	Credible interval
σ^2^_u_	Additive genetic variance
σ^2^_e_	Residual variance
σ^2^_m_	Maternal variance
