# Supplementary material for: The Role of Genetic and Environmental Factors in White Leg Markings: Prevalence and Heritability Analysis in Pura Raza Española Horses
Source: Life (Basel). 2025 Oct 23;15(11):1661. doi: 10.3390/life15111661 (PMC12653778; doi:10.3390/life15111661)
Supplement: Supplementary file 1 [file life-15-01661-s001.zip › life-3923266-supplementary.pdf]

Supplementary Table S1. Bayesian generalized multivariate (non-)linear multilevel models and "relevant" level (when the 95% credible interval does not include zero and are highlighted in grey) between white markings in leg score (Discrete scale) and systematic risk factors and tests for comparing proportions between groups for discrete systematic risk factors.

Leg	Class	Sex		Coat Colour				Imbreeding	Maternal effect
		Male	Female	Bay	Chestnut	Black	Grey	(95% CI)	SD (95% CI)
LF	0	93.26[a]	95.45[b]	95.07[c]	91.98[a]	96.57[d]	94.36[b]	(0.18, 2.25)	(0.95, 1.22)
	1	1.02[b]	0.77[a]	0.91[a]	1.00[a]	0.72[a]	0.81[a]		
	2	4.41[b]	3.03[a]	3.22[b]	4.37[c]	2.25[a]	3.97[c]		
	3	1.31[b]	0.74[a]	0.80[b]	2.64[c]	0.45[a]	0.85[b]		
RF	0	94.11[a]	95.99[b]	95.86[c]	92.68[a]	97.S25[d]	94.85[b]	(-0.06, 2.25)	(0.83, 0.99)
	1	0.90[a]	0.73[a]	0.80[a]	0.82[a]	0.56[a]	0.84[a]		
	2	3.86[b]	2.59[a]	2.70[b]	3.61[c]	1.84[a]	3.56[c]		
	3	1.13[b]	0.69[a]	0.64[b]	2.89[c]	0.35[a]	0.76[b]		
LH	0	79.39[a]	83.42[b]	80.52[b]	77.55[a]	83.10[c]	84.14[c]	(-0.61, 0.71)	(0.85, 0.99)
	1	2.45[b]	2.04[a]	2.46[b]	1.52[a]	3.00[c]	1.79[a]		
	2	12.95[b]	10.70[a]	12.34[b]	11.12[ab]	10.95[a]	10.85[a]		
	3	5.21[b]	3.84[a]	4.67[b]	9.81[c]	2.95[a]	3.21[a]		
RH	0	82.94[a]	86.80[b]	85.01[b]	80.83[a]	86.61[c]	86.58[c]	(-0.38, 1.08)	(0.83, 1.00)
	1	2.00[a]	1.76[a]	2.10[b]	1.52[a]	2.27[b]	1.52[a]		
	2	10.96[b]	8.38[a]	9.45[a]	9.26[a]	8.90[a]	9.18[a]		
	3	4.10[b]	3.06[a]	3.44[b]	8.38[c]	2.21[a]	2.72[a]		

LF: Left foreleg; RF: Right foreleg; LH: Left hindleg; RH: Right hindleg; class 0: unaffected; class 1: white markings up to the cannon bone; class 2: markings below the fetlock; class 3: markings above the fetlock; CI: 95% Credible Interval; SD: Standar desviation

Supplementary Table S2. Bayesian generalized multivariate (non-)linear multilevel models and "relevant" level (when the 95% credible interval does not include zero and are highlighted in grey) between white markings in leg score (Dichotomous trait) and systematic risk factors and tests for comparing proportions between groups for discrete systematic risk factors.

Leg	Markings	Sex		Coat Colour				Imbreeding	Maternal effect
		Male	Female	Bay	Chestnut	Black	Grey	(95% CI)	SD(95% CI)
LF	Without	93.26^{a}	95.45^{b}	95.07^{c}	91.98^{a}	96.57^{d}	94.36^{b}	(0.25, 2.27)	(0.91, 1.19)
	With	6.74^{b}	4.55^{a}	4.93^{b}	8.02^{d}	3.43^{a}	5.64^{c}		
RF	Without	94.11^{a}	95.99^{b}	95.86^{c}	92.68^{a}	97.25^{d}	94.85^{b}	(0.02, 2.23)	(0.99, 1.28)
	With	5.89^{b}	4.01^{a}	4.14^{b}	7.32^{d}	2.75^{a}	5.15^{c}		
LH	Without	79.39^{a}	83.42^{b}	80.52^{b}	77.55^{a}	83.10^{c}	84.14^{c}	(-0.62, 0.74)	(0.85, 1.00)
	With	20.61^{b}	16.58^{a}	19.48^{b}	22.45^{c}	16.90^{a}	15.86^{a}		
RH	Without	82.94^{a}	86.8^{b}	85.01^{b}	80.83^{a}	86.61^{c}	86.58^{c}	(-0.46, 0.93)	(0.82, 0.98)
	With	17.06^{b}	13.20^{a}	14.99^{b}	19.17^{c}	13.39^{a}	13.42^{a}		
A	Without	96.98^{a}	98.06^{b}	98.06^{c}	95.87^{a}	98.57^{d}	97.44^{b}	(0.43, 3.39)	(1.03, 1.46)
	With	3.02^{b}	1.94^{a}	1.94^{b}	4.13^{d}	1.43^{a}	2.56^{c}		
B	Without	88.89^{a}	91.86^{b}	90.64^{b}	87.06^{a}	91.86^{c}	91.54^{c}	(-0.87, 0.83)	(0.86, 1.09)
	With	11.11^{b}	8.14^{a}	9.36^{b}	12.94^{c}	8.14^{a}	8.46^{a}		
C	Without	95.46^{a}	97.02^{b}	96.8^{c}	94.35^{a}	97.85^{d}	96.22^{b}	(0.09, 2.60)	(0.96, 1.30)
	With	4.54^{b}	2.98^{a}	3.20^{b}	5.65^{d}	2.15^{a}	3.78^{c}		
D	Without	96.36^{a}	97.62^{b}	97.55^{c}	95.11^{a}	98.49^{d}	96.89^{b}	(-0.15, 2.74)	(1.03, 1.43)
	With	3.64^{b}	2.38^{a}	2.45^{b}	4.89^{c}	1.51^{a}	3.11^{c}		
E	Without	96.04^{a}	97.43^{b}	97.44^{c}	94.84^{a}	98.04^{d}	96.61^{b}	(-0.04, 2.62)	(0.86, 1.24)
	With	3.96^{b}	2.57^{a}	2.56^{b}	5.16^{d}	1.96^{a}	3.39^{c}		
F	Without	96.01^{a}	97.35^{b}	97.26^{c}	95.02^{a}	98.2^{d}	96.54^{b}	(-0.51, 2.15)	(0.87, 1.27)
	With	3.99^{b}	2.65^{a}	2.74^{b}	4.98^{d}	1.80^{a}	3.46^{c}		
G	Without	98.40^{a}	98.93^{b}	99.03^{c}	97.39^{a}	99.15^{c}	98.65^{b}	(-0.84, 3.22)	(0.96, 1.62)
	With	1.60^{b}	1.07^{a}	0.97^{a}	2.61^{c}	0.85^{a}	1.35^{b}		

LF: Left foreleg; RF: Right foreleg; LH: Left hindleg; RH: Right hindleg; A: Left foreleg + Right Foreleg: (LF) + (RF); B. Left hindleg + Right hind leg: (LH) + (RH); C. Left legs: (LF) + (LH); D. Right legs: (RF) + (RH); E. Left foreleg + Right hind leg: (LF) + (RH); F. Right Foreleg + Left hind leg: (RF) + (LH); G. Four legs: (LF) + (RF) + (LH) + (RH); CI: 95% Credible Interval; SD: Standar desviation.
